# Molecular Evolution of Calcium Signaling and Transport in Plant Adaptation to Abiotic Stress

**DOI:** 10.3390/ijms222212308

**Published:** 2021-11-15

**Authors:** Tao Tong, Qi Li, Wei Jiang, Guang Chen, Dawei Xue, Fenglin Deng, Fanrong Zeng, Zhong-Hua Chen

**Affiliations:** 1 Hubei Collaborative Innovation Center for Grain Industry, College of Agriculture, Yangtze University, Jingzhou 434022, China; 202073058@yangtzeu.edu.cn (T.T.); 201871363@yangtzeu.edu.cn (W.J.); dfl@yangtzeu.edu.cn (F.D.); 2 Central Laboratory, Zhejiang Academy of Agricultural Science, Hangzhou 310030, China; qili0605@zju.edu.cn (Q.L.); chenguang0915@zju.edu.cn (G.C.); 3 College of Life and Environmental Sciences, Hangzhou Normal University, Hangzhou 311121, China; dwxue@hznu.edu.cn; 4 School of Science, Western Sydney University, Penrith 2751, Australia; 5 Hawkesbury Institute for the Environment, Western Sydney University, Penrith 2751, Australia

**Keywords:** calcium ion, phylogenetic analysis, abiotic stress, ion transport, regulatory network

## Abstract

Adaptation to unfavorable abiotic stresses is one of the key processes in the evolution of plants. Calcium (Ca^2+^) signaling is characterized by the spatiotemporal pattern of Ca^2+^ distribution and the activities of multi-domain proteins in integrating environmental stimuli and cellular responses, which are crucial early events in abiotic stress responses in plants. However, a comprehensive summary and explanation for evolutionary and functional synergies in Ca^2+^ signaling remains elusive in green plants. We review mechanisms of Ca^2+^ membrane transporters and intracellular Ca^2+^ sensors with evolutionary imprinting and structural clues. These may provide molecular and bioinformatics insights for the functional analysis of some non-model species in the evolutionarily important green plant lineages. We summarize the chronological order, spatial location, and characteristics of Ca^2+^ functional proteins. Furthermore, we highlight the integral functions of calcium-signaling components in various nodes of the Ca^2+^ signaling pathway through conserved or variant evolutionary processes. These ultimately bridge the Ca^2+^ cascade reactions into regulatory networks, particularly in the hormonal signaling pathways. In summary, this review provides new perspectives towards a better understanding of the evolution, interaction and integration of Ca^2+^ signaling components in green plants, which is likely to benefit future research in agriculture, evolutionary biology, ecology and the environment.

## 1. Introduction

Plants have to cope with constantly changing environments, which are often stressful for plant growth, development and reproduction [1]. Abiotic stresses in adverse environmental conditions such as drought, extreme temperature, nutrient deficiency, salinity, and toxic metal(loid)s, are regarded as major environmental factors that affect the geographical distribution of plants in nature and limit the productivity and quality of agricultural crops [2,3]. Plants can sense abiotic stresses through specific sensors and integrated signaling pathways (e.g., Calcium (Ca^2+^) signaling) to alter their physiology, metabolism and development [1]. Therefore, improving the abiotic stress tolerance of plants is essential for global food security and environmental sustainability. 

Calcium is an essential macronutrient for plant growth and development, and acts as a crucial second messenger in regulating plant responses to abiotic stresses in plants. Abiotic stresses can induce an increase in spatiotemporal variations of intracellular Ca^2+^ concentration in plant cells, which is one of the vital adaptive strategies in response to developmental cues and external stimuli, such as gravity, nutrient status, mechanical stimulation, temperature shifts, light, salinity, drought, heavy metals, and metalloids [4,5,6,7,8]. As a whole system, calcium signaling is composed of conserved multidomain proteins that regulate diverse signaling events, contributing to the evolution of abiotic stress tolerance from ancestral to extant plants [9,10,11]. Recent studies showed that, in comparison to those in animals, some components of Ca^2+^ signaling during plant evolution have diversified, while others have either been lost or have not evolved in some plant lineages. For instance, plants may have lost diversity in plasma membrane calcium-influx mechanisms, which can be compensated for by the evolution of the function of the calcium-storing vacuole. It was thus proposed that plants may use a system based on intracellular second messengers to adapt to changing environmental conditions [12].

**Figure 1:**
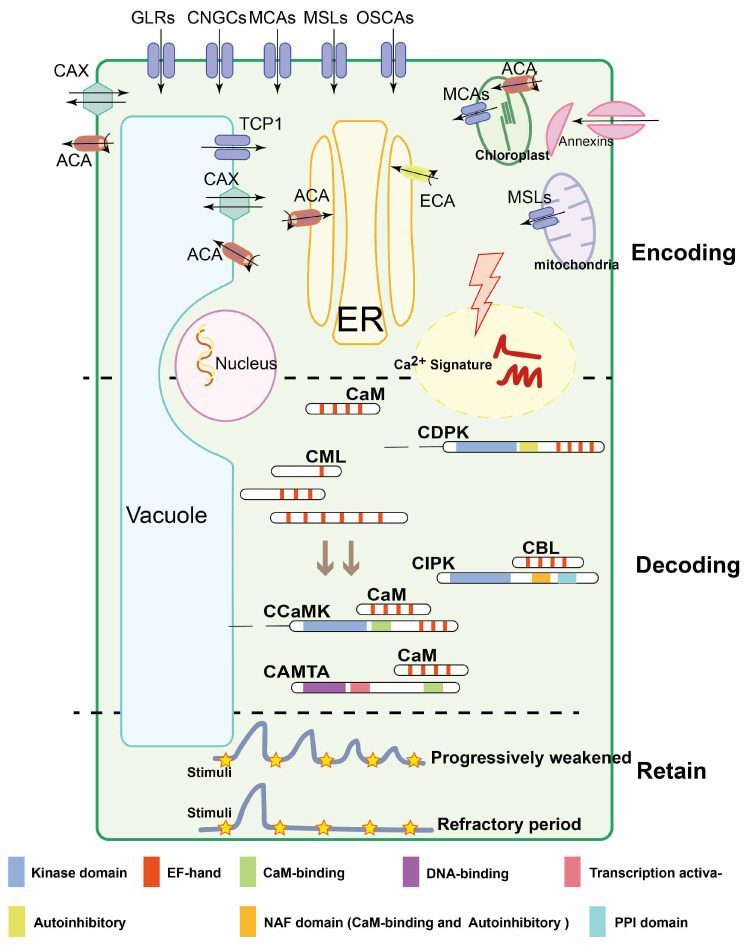
Three major processes implicated in Ca^2+^ signal stimulus-induced dynamic alterations: encoding, decoding and retaining. Ca^2+^ encoding is mediated by Ca^2+^ channels, Ca^2+^-ATPases, Ca^2+^/H^+^ exchangers, channels named CNGCs, GLRs, TPCs, MCAs, MSLs, OSCA1, ATPases named ACAs, ECAs, and the antiporter named CAX; besides this, annexins without EF-hand domains are also involved in Ca^2+^ influx. The decoding is composed of many different protein families. CaMs, CMLs and CBLs harbor EF hand domains and regulate target proteins without any additional functional domains. CBLs modulate the activity of CIPKs interacting with NAF and PPI domain, while CDPKs are directly activated by Ca^2+^ binding to the CaM-like domain. In contrast to CDPKs, CCaMKs are dual-regulated kinases. These proteins bind Ca^2+^ via a visinin-like domain, while in addition, Ca^2+^-CaM binds to the regulatory domain of the kinase and mediates further activation. CAMTAs activate the transcription of related genes by interacting with CaM. Ca^2+^ signatures retain a steady-state concentration under the same stimulation for a short period through persistent decaying or during a refractory period. Colored modules represent different functional structures. The black lines with an arrow indicate the direction of Ca^2+^ flow, and the black curve with an arrow indicates the ATP decomposition reaction (ATP to ADP).

Ca^2+^ can undergo dynamic alterations in spatiotemporal concentration according to the type and intensity of a specific response to a stimulus [13]. A distinct set of Ca^2+^ changes defined by their amplitude, frequency and duration is referred to as a ‘Ca^2+^ signature’, containing transient calcium, calcium oscillation, calcium signal space orientation, and calcium waves. These perturbations can be distinguished by coordination between Ca^2+^ influx and transport at specific subcellular locations, and the kinetics of its magnitude [4,14]. For encoding and decoding the information of specific Ca^2+^ signatures, plants are equipped with many Ca^2+^ transporters and intracellular sensor proteins. Calcium channels, pumps and transporters regulate the electrochemical potential. Large families of Ca^2+^ sensor proteins in plants contain E and F regions of parvalbumin (EF hand) that are represented by calmodulins (CaMs) and calmodulin-like proteins (CMLs), Ca^2+^-dependent protein kinases (CDPKs) and CDPK-related kinases (CRKs), and calcineurin B-like proteins (CBLs) and CBL-interacting protein kinases (CIPKs) (Figure 1) [14,15]. Our understanding of calcium signaling has been driven by research at the level of individual genes to the cellular level. Multifaceted in-depth investigation of Ca^2+^-signaling specificity using molecular cloning, transgenic approaches, bio-imaging, bioinformatics, CRISPR-Cas9-mediated gene editing, interaction networks screening and omics have revealed the biological relevance of intracellular Ca^2+^-signaling networks with the external environment [6,16,17,18,19].

However, a review of the evolution of Ca^2+^ signaling systems across the major lineages of green plants is still lacking, especially in the context of abiotic stresses. This review focuses on the dynamic properties of Ca^2+^ signaling and its complex molecular network from environmental interaction, cellular function and evolutionary perspectives. Readers are also directed to existing excellent reviews for a more systematic overview of calcium signaling [6,20,21,22].

## 2. Calcium Signal Transduction in Responses to Abiotic Stress

Plants can adaptively respond to the harmful effects of abiotic stresses by coordinating various Ca^2+^-sensing molecules, generating macroscopic phenotypic divergence [23,24]. Many abiotic stresses induce the generation of calcium signals in the cytoplasm and transmit the signal downstream by interacting with Ca^2+^-binding protein—thus activating downstream gene expression events and related ion transport activities, leading to other downstream cellular responses [21,25]. Here, we summarize the responses of Ca^2+^ signaling in plants to a few major abiotic stresses including drought, salinity, and extreme temperature.

### 2.1. Multiplicity of Abiotic Stresses and the Role of the Ca^2+^-Sensing Network

In plants, drought stress is closely associated with osmotic stress, and detecting it involves plasmolysis, plasma membrane depolarization, and damage to the plasma membrane and cell wall [26]. Among the Ca^2+^ sensors for osmotic stress, arabidopsis reduced hyperosmolality-induced [Ca^2+^]_i_ increase 1 (AtOSCA1) encodes a plasma membrane calcium-permeable channel, which is responsible for the hyperosmolality-induced transient elevation in Ca^2+^ [27]. Thus, AtOSCA1 affects the generation of stretch force on the plasma membrane and membrane–cell wall interactions by reducing cell turgor [28]. Calcium-permeable stress-gated cation channels (CSCs) have been identified as paralogs of OSCAs, which are also recognized as candidates for osmo- or mechano-sensitive Ca^2+^ signaling processes in plants (Figure S1) [29]. Moreover, Arabidopsis mechanosensitive-like channel 8 (AtMSL8) is required for pollen survival through modulation of hypotonic-induced membrane tension under water deficit-induced osmotic stress [30]. In rice (*Oryza sativa*), a novel small calcium-binding protein, OsCCD1, harboring one EF-hand motif was reported to enhance tolerance to osmotic stress through calcium-mediated abscisic acid (ABA) signaling [31]. Similarly, loss-of-function in AtCDPK21/23 can instead improve the tolerance to hyperosmotic stress in Arabidopsis mutants [32,33]. Overall, rapid Ca^2+^ rises triggered by these osmotic sensors usually correlate with induction changes in cell membrane tension.

Under salt stress, it is well-established that plants employ a calcium-dependent salt-overly-sensitive (SOS) pathway to mediate signal transduction [34]. The EF calcium-binding protein SOS3/CBL4 senses salt stress-mediated cytoplasmic Ca^2+^ signals; SOS3 cooperates with SOS2/CIPK24 to induce phosphorylation and activation of SOS1/NHX7, a plasma membrane Na^+^/H^+^ transporter [34,35,36]. In Italian millet (*Setaria italica*), the SiCBL5-SiCIPK24-SiSOS1 pathway is involved in salt tolerance by regulating Na^+^ homeostasis [37]. This Ca^2+^-SOS3-SOS2-SOS1 module suggests that the signaling module combining CBL-CIPK-transporters may be ubiquitously utilized for adapting to salinity and other abiotic stresses in plants (Figure S1). For instance, intracellular potassium (K^+^) homeostasis is critical for plant survival in saline environments [38]. Low K^+^ stress possibly triggers cytoplasmic Ca^2+^ signaling through the activation of AtCIPK23 by AtCBL1 and AtCBL9, which phosphorylates and activates the potassium channel Arabidopsis K transporter 1 (AKT1) [39,40,41]. In rice, OsCBL1 and OsCIPK23 modules maintain a stable K^+^ concentration in root cells [42]. AtCBL2 and AtCBL3 redundantly interact with the proteins AtCIPK3/9/23/26 to regulate Mg^2+^ distribution in vacuoles and form tolerance to high Mg^2+^ stress [43]. Moreover, CDPK21 functions as an intermediate regulation node of the outwardly rectifying K^+^-channel GORK and 14-3-3 proteins [44], and CDPK13 specifically phosphorylates the guard cell K^+^ influx channels, KAT1 and KAT2 [45]. Therefore, a combination based on CBL-CIPK modules performs with more versatility and flexibility, particularly in the regulation of various abiotic signals that mediate ion transport.

Temperature fluctuations can impose various complex effects on plant cells through key components of Ca^2+^ signaling [46,47]. In Arabidopsis, cold stress is proposed to be sensed by the Ca^2+^-permeable mechanosensitive channels MCA1 and MCA2 (mid1-complementing activity), and regulated through membrane fluidity and cytoskeletal reorganization. This leads to calcium influx and activation of CDPKs, CBLs-CIPKs, and mitogen-activated protein kinases (MAPK) that mediate cold-responsive (COR) gene expression [47]. Additionally, chilling tolerance divergence 1 (OsCOLD1), a transmembrane protein of the plasma membrane and endoplasmic reticulum (ER) that regulates calcium channels in rice, is a potential cold stress sensor for resistance to chilling damage (Figure S1) [48]. The Arabidopsis calcium exchanger 1 (AtCAX1), encoding a vesicle membrane Ca^2+^/H^+^ antiporter, is involved in the developmental process of the low-temperature response process [49]. Moreover, a plasma membrane cyclic nucleotide-gated ion channel (CNGC) OsCNGC9 triggers Ca^2+^ elevation to enhance chilling tolerance in rice [50]. Ca^2+^/CaM-regulated receptor-like kinases 1 (CRLK1) and calmodulin-binding transcriptional activator 3 (CAMTA3) are both involved in plant responses to cold stress [51]. For heat stress, OsCNGC14 and OsCNGC16 function as modulators of cytosolic calcium uptake in rice [52] and LeCDPK2 can effectively protect plants from heat stress in tomato [53]. AtCaM3 can interact with CaM-binding protein kinase or mediate heat-activated mitogen-activated protein kinase [54], followed by the phosphorylation of heat shock factors (HSFs) and the expression of heat shock proteins (HSPs), ultimately leading to the heat shock response and enhanced thermotolerance (Figure S1) [55]. Recently, high temperature sensitive 1 (HTS1), a β-Ketoacyl carrier protein reductase, was identified as regulating the transcriptional activity of HSFs and HSPs [46]. In conclusion, heat and cold stresses usually alter the integrity of membranes and Ca^2+^ signaling pathways, causing alterations in the expression of a range of downstream temperature-related factors in plants.

### 2.2. Calcium-Mediation of Hormonal Signaling

Abiotic stress resistance in plants can be enhanced via the control of hormonal signaling pathways where Ca^2+^ is a crucial regulatory node. Here, we use the well-documented ABA and auxin signaling as examples. ABA-regulated stomatal movement utilizes Ca^2+^ signaling, which is dependent on the Pyrabactin resistance/Pyrabactin resistance-like/regulatory component of the ABA receptor (PYR/PYL/PCAR)–protein phosphatase 2C (PP2C)–sucrose non-fermenting 1-related protein kinase 2 (SnRK2) core pathway (Figure S1) [56,57]. In this process, ABA regulates the PM-localized Ca^2+^ channels through the generation of reactive oxygen species (ROS), which was reduced in the *pyr1*/*pyl1*/*pyl2*/*pyl4* mutant [58]. Moreover, AtCDPK4/5/11/12/32, as the alternative stomatal regulatory pathways independent of SnRK2s, are proposed to interact with ABA-responsive transcription factors (TFs) such as ABF1 and ABF4, and modulate downstream anion channels in Arabidopsis [16,56,57,59,60,61,62]. These findings indicate that CDPKs enhance vegetative resistance or tolerance to drought and high salinity by regulating ABA signaling pathways in plants [62]. In addition, ABA-induced calcium signaling can also activate the AtCBL1/9-AtCIPK26 module, leading to phosphorylation of effector proteins such as respiratory burst oxidase homologue (RBOH), resulting in the generation of ROS [63]. Moreover, CaMs and CMLs function as the characterized Ca^2+^ sensors implicated in multiple abiotic stresses through interaction with target proteins (Figure S1) [64]. AtCML9/24/37 positively modulate ABA-induced vegetative stress responses such as drought and salt [65,66,67,68]. Noteworthily, Ca^2+^ transients are elicited on exposure to auxin through the collaboration of the Ca^2+^-permeable channels AtCNGC14 and AtCAX1 [69,70]. As a Ca^2+^ sensor, CaCIPK6 exhibits novel functions in root development via alteration of auxin transport [71]. Comparatively, AtCDPK3/4 modify auxin signal transduction by phosphorylating phospholipase A (PLA; Figure S1) [72,73]. Auxin also can elicit specific Ca^2+^ signaling patterns to regulate the expression of AtCML1 [74]. However, the roles of Ca^2+^ signaling in the pathways of the other seven major phytohormones still require further investigation and are not reviewed here.

Cross regulation exists between multiple phytohormone signaling pathways and Ca^2+^ signaling components, which form an intricate signaling regulatory network [73]. The regulation of stomatal opening and closure mediated by CDPKs often require synergism between various phytohormones. For instance, ABA and jasmonic acid (JA) interaction is connected by CDPK6 because CDPK6 and CDPK3 cooperatively regulate ABA-mediated stomatal closure, whereas JA-mediated stomatal movement displays exclusive reliance on CDPK6 [75,76]. In rice, the expression of *OsCDPK13* was upregulated in response to gibberellic acid (GA) while it was inhibited by ABA [73] and AtCDPK28 negatively regulates the balance of JA and GA, which is crucial for plant defense and development [77]. Therefore, CDPKs, combining a Ca^2+^-sensing domain and a kinase catalytic domain, are major contact nodes for interaction with phytohormones, which provide the molecular basis for the establishment of regulatory networks (Figure S1) for future research endeavors.

## 3. Calcium Dynamics in Plant Cells

### 3.1. Transporters Shape the Ca^2+^ Signature

The dynamics of intracellular Ca^2+^ have been extensively investigated in plants. The polar growth of pollen tubes and stomatal closure can be triggered by Ca^2+^ oscillations [59,78], allowing Ca^2+^ influx into the cytoplasm; subsequently, these specific and asymmetric patterns of oscillations elicit physiological responses [79,80,81]. As the initial plant response signal, Ca^2+^ needs selective transport by channels, pumps, and transporters localized on the membranes, based on experimental and modelling approaches [4,80,81,82]. For example, Ca^2+^-permeable channels lead to a rapid elevation of cytosolic Ca^2+^, and Ca^2+^-ATPases, and Ca^2+^/H^+^ exchangers remove cytosolic Ca^2+^ according to the electrochemical gradient [83].

Ca^2+^-permeable voltage-gated and ligand-gated channels are usually selective to mono- and divalent cations in plant cells. These channels that are localized to various organelle membranes can be activated by electrochemical potentials or ligand binding, such as glutamic acid (Glu), inositol triphosphate (IP3), cyclic ADP ribose (cADPR) and cyclic nucleotide monophosphate (cNMPs) [83,84]. The recent functional annotation and experimental analysis of Ca^2+^-permeable channels provided useful clues for their involvement in generating calcium signals (Figure 1). Mechanosensitive channels, including MSLs and MCAs, trigger Ca^2+^ signatures with mechanical osmotic stimuli [83]. OSCA1 and the nonselective cation channels (NSCCs) can be activated by hyperosmolality to interfere with Ca^2+^ fluxes [27,29]. In addition, two pore calcium channel 1 (TPC1), located in the tonoplast, provides Ca^2+^- and voltage-dependent Ca^2+^ release from vacuoles to regulate abiotic stress responses in important cell types such as the stomatal guard cells (Figure 1) [85]. Calcium efflux from the cytosol drives the redistribution of Ca^2+^ between the symplast and apoplast, and returns the electrochemical potential back to resting Ca^2+^ levels, which may contribute to shaping the specific and distinct calcium signatures. Ca^2+^-ATPases and Ca^2+^/H^+^ antiporters are the pivotal proteins catalyzing this process (Figure 1). Ca^2+^-ATPases are composed of the endoplasmic reticulum (ER)-type Ca^2+^-ATPases (ECA or type IIA) and the auto-inhibited Ca^2+^-ATPases (ACA or type IIB); the expression of several ACAs and ECAs can be induced by salt stress in barley root [86] and waterlogging responses in Arabidopsis [87]. AtCAX1 regulates chilling responses and metal hypersensitivity via sequestering of Ca^2+^ into the vacuole [88,89]. However, these studies mainly focused on the detailed molecular function of individual Ca^2+^ transporters in abiotic stresses. We propose that future research work should consider the interaction of those key Ca^2+^ transporters with other key components of Ca^2+^ signaling in different types of cells to realize their fundamental role in plant abiotic stress tolerance.

### 3.2. Ca^2+^-Signaling Sensors

Any modification in the concentration of Ca^2+^ is subsequently decoded in the targeted cells to induce appropriate responses depending on the types and levels of abiotic stresses, where calcium sensors play vital roles in this process. Calcium sensors are divided into three groups: sensor relays (e.g., CaMs, CMLs, and CBLs), sensor protein kinases (e.g., CDPKs), and bimolecular sensor responders (e.g., calmodulin-binding transcription activators (CAMTAs), Ca^2+^-CaM-dependent kinases (CCaMKs), and CIPKs (Figure 1) [90,91,92]. Here, we summarize the functions of these Ca^2+^ sensors in plant abiotic stress tolerance.

#### 3.2.1. Calmodulins and Calmodulin-Dependent Proteins

CaMs are highly conserved Ca^2+^-dependent regulatory proteins composed of two globular domains with two EF-hands for Ca^2+^-binding [14,93]. Due to the lack of kinase activity, CaMs change into an active conformation only after modification with Ca^2+^ binding, which allows interaction with proteins [94]. This interaction subsequently activates or inhibits target proteins [95,96], translating a Ca^2+^ signal into a molecular response (Figure 1). Arabidopsis has 7 CaMs and 47 CMLs, which have a certain degree of homology to CaMs [11]. CMLs exhibit high divergence in their number of EF-hand motifs (1 to 6) [97], diverse subcellular localization and tissue-specific expression [98]. For example, AtCML30 and AtCML3 are targeted to mitochondria and peroxisomes in Arabidopsis, respectively [99].

Plant calmodulin-dependent protein kinases (CaMKs) are activated or enhanced by binding with specific CaMs and there are CaMKs that harbor a CaM-binding domain in some plant species (Figure 1) [100,101]. Some receptor-like protein kinases localized on the plasma membrane and cytoplasm are also activated through interactions with Ca^2+^/CaM. For instance, with the presence of Ca^2+^/CaM, AtCRLK1 modulates cold acclimation through a MAP kinase cascade in Arabidopsis [102]. Calmodulin-binding transcription activators (CAMTAs), one interacting partner of CaMs, can be found from the major TF families, such as the basic leucine zipper (bZIP), WRKY and myeloblastosis (MYB) families [103,104]. These TFs play critical roles in signaling and the response to abiotic stresses through the regulation of downstream target genes [26,104,105]. For instance, HvCaM1 is transcriptionally regulated by HvCAMTA4, and then co-modulates K^+^ channels to alleviate salt stress in barley shoot [106]. Taken together, these studies demonstrated that Ca^2+^/CaMs are one of the crucial nodes for the transcriptional regulation of downstream genes in the Ca^2+^ signaling of abiotic stresses.

#### 3.2.2. Calcium-Dependent Protein Kinases

Classical CDPKs have a calcium-binding domain (CBD), a serine/threonine protein kinase domain (PKD), an autoinhibitory junction (AJ), and an N-terminal variable domain (NTD) [14]. CDPKs are activated by an increase in cytosolic Ca^2+^ concentration through the translocation of an autoinhibitory domain and a conformational change resulting from the interaction between Ca^2+^ and the binding domain (Figure 1) [107,108]. In Arabidopsis, there are 34 CDPK members [107], which can specifically bind to the plasma membrane, endoplasmic reticulum membrane and peroxisome membrane after acylation [97], providing a functional foundation to exquisitely regulate the activity of target proteins near their binding sites. CDPKs are also required for the translation of mobile signals to distant tissues through the rapid propagation of Ca^2+^ waves up to 400 µM/s [20,109]. Certainly, the generation of Ca^2+^ waves depends on the Ca^2+^-permeable channels of the endomembranes, such as the vacuolar TPC1 and ER-localized GLR3.1 [15,110]. Respiratory burst oxidase homologue of D (RBOHD) can achieve rapid ROS-mediated signaling in response to abiotic stresses [22,111,112,113]. Intriguingly, CDPK5 phosphorylates RBOHD, and simultaneously, its activity is mediated by ROS; ultimately, these reactions construct the circuit of self-propagating mutual activation and feed-forward amplification [17,114]. Furthermore, several CDPKs, and other proteins such as 14-3-3 proteins, can interact and phosphorylate H^+^-ATPases to generate multiple electrical signals in abiotic stress responses [115,116,117]. Thus, antagonistic roles between CDPKs and other Ca^2+^ sensor proteins may implicate a fine-tuning of the flow of Ca^2+^ during signaling transduction [14]. CRKs display structural domains similar to CDPKs, but with a degenerative C-terminal CaM-like regulatory domain (CaMLD). Thus, the Ca^2+^-dependent manner of CRKs is binding with CaMs rather than being directly regulated by Ca^2+^ [118]. For example, one of the functions of CRKs is the positive regulation of root growth and gravitropism via establishment of the proper auxin gradient and modulation of polar auxin transport (PAT) proteins [119,120,121]. In short, CDPK subfamilies are vital regulatory nodes in Ca^2+^ signaling pathways and abiotic stress in plants.

#### 3.2.3. The CBL–CIPK Signaling Network

There are 10 CBLs in both Arabidopsis and rice [122], while 26 and 30 CIPKs are presented in the genome of Arabidopsis and rice, respectively [123,124]. CBLs usually only harbor one EF-hand for Ca^2+^ binding without enzymatic activity. The different number of EF-hand domains in members of the CBL family suggests different capacities and affinities in their specific roles in the decoding of Ca^2+^ signal in plants (Figure 1) [108]. Importantly, CBL proteins sense Ca^2+^ signatures through four EF-hands and interact with the CBL-binding domain of CIPKs—the C-terminal asparagine–alanine–phenylalanine (NAF)-domain [92,125]. The NAF domain is also required for releasing the kinase domain and thereby transforms the N-terminal kinase domain of CIPKs into an active conformation via the binding of CBL proteins [123]. In addition, CIPKs can further amplify the activation signal through autophosphorylation and transphosphorylation of the activation loop in the kinase domain [71]. CBL-CIPK interactions are determined by several factors, including structural differences in CBL, differences in the NAF region of CIPK and the sequences on either side of it [126]. Thus, the complexity and diversity of the CBL-CIPK signaling system and the spatial specificity of its target identification are determined by their interplay features [122,127], which allow a plant to fine-tune its response to abiotic stress, via both pre- and post-translational mechanisms. In *Arabidopsis*, drought, salinity, and low temperature all induced *CBL* and *CIPK* gene expression [71,124]. In rice, among the 31 OsCIPKs, 20 were induced by abiotic stresses such as drought, cold, and salinity [128,129]. In summary, the CBL-CIPK regulatory module represents one of the critical components of the Ca^2+^ signaling pathway for plant adaptation to fluctuating environments.

#### 3.2.4. Ca^2+^ Binding Proteins without EF-Hands

There are several Ca^2+^-binding proteins including the annexins (ANN) [130], phospholipase D (PLD) [131], calreticulin (CRT) [132], calnexin (CNX) [133] and pistil-expressed Ca^2+^-binding protein (PCP) [134], which do not contain EF-hand motifs. Among them, the annexins are a highly conserved protein family known to be associated with Ca^2+^, membrane phospholipids, cytoskeletal components, and ATPase and peroxidase activities in plants (Figure 1). Structurally, plant annexins are composed of four repetitive regions, among which repeat 1 and 4 possess elevated conservation from unique internal fusion amino acid residues [135,136]. The molecular functional analysis of annexins is starting to provide further insights. Arabidopsis AtANN1 and AtANN4 function cooperatively to regulate drought and salt stress responses in a Ca^2+^-dependent manner [137], and AtANN1 also acts as a pH-sensitive Ca^2+^-permeable transporter in response to environmental stimuli [138,139]. However, the exact roles for plant ANNs in Ca^2+^ signaling either as putative Ca^2+^ sensors or Ca^2+^ transporters are still being investigated.

### 3.3. Ca^2+^ Signature Memory for Abiotic Stresses

Based on considerable evidence, it has been proposed that plants possess a Ca^2+^ “memory” by which cytosolic Ca^2+^ signatures can recognize and be specifically modified according to previous experiences with abiotic stresses [140,141]. The diminished response of cytosolic Ca^2+^ after repeated stimulation by the same abiotic stress forms part of this cellular memory and the cells are able to retain previous information (Figure 1) [142]. This Ca^2+^ memory resembles a balance of Ca^2+^ levels for a better response to particular abiotic stresses, rather than disturbing the delicate Ca^2+^ balance in different parts of a plant cell. For instance, the magnitude of the Ca^2+^ perturbation caused by wind-induced mechanical stress becomes progressively smaller after repeated stimulation and requires several minutes before a complete Ca^2+^ response is observed again. Similarly, Ca^2+^ memory is one of the strategies for plant adaptation to heat stress via the acquisition of thermal memory during pre-exposure to sublethal heat stress [143]. Moreover, *Arabidopsis* plant cells failed to respond to H_2_O_2_ (a key molecule in the Ca^2+^ signaling pathway) for several hours under challenge with H_2_O_2_ treatment. These plants were more resistant to cold stress and generally retained Ca^2+^ memory more often than untreated plants [144]. These findings again strengthen the important links between Ca^2+^ signaling and abiotic stress tolerance in plants.

## 4. Evolution of Calcium Signaling for Abiotic Stresses in Green Plants

Ca^2+^ signaling systems fulfill the role of signal transduction in organisms ranging from aquatic unicellular algae to terrestrial multi-cellular higher plants [145]. Ca^2+^ signaling systems are in constant reform, expanding and diversifying enormously to adapt to the changing external environment—especially in abiotic stresses [146]. Consequently, it is of importance to trace the molecular function of Ca^2+^ transporters and sensors to obtain crucial evolutionary insights.

### 4.1. Comparative Genetic and Evolutionary Analysis of Calcium-Related Gene Families

**Figure 2:**
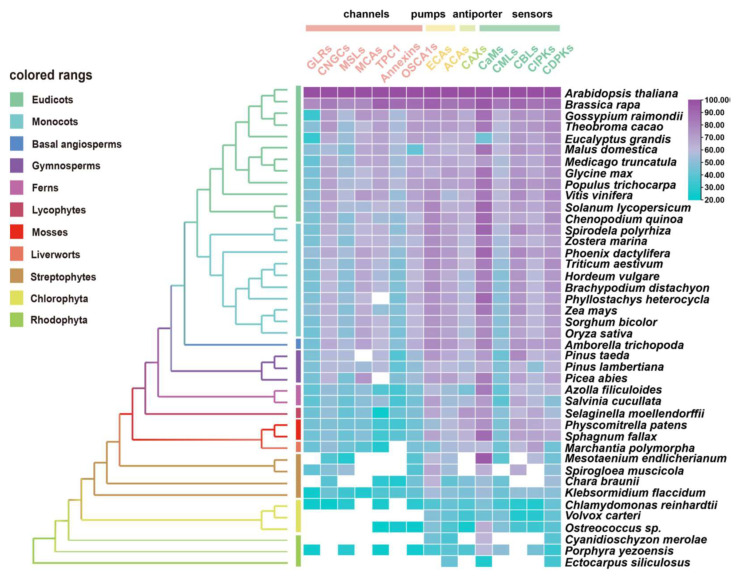
Similarity heat map for the evolution of Ca^2+^ signaling relative proteins, containing channels, pumps, antiporters, and sensors in different plant and algal species. Candidate protein sequences were selected by BLASTP searches which satisfied E value < 10^−10^ and query coverage > 50% [7]; colored squares indicate a protein sequence similarity from 20% (blue) to 100% (purple). White squares indicate proteins that satisfied neither of the selection criteria. GLRs, glutamate receptor-like channels; CNGCs, cyclic nucleotide gated channels; MSLs, mechanosensitive-like channels; MCAs, ‘mid1-complementing activity’ channels; TPC1, two-pore channel 1; OSCA1s, hyperosmolality-induced Ca^2+^ channel 1; ECAs, endoplasmic reticulum-type Ca^2+^-ATPases; ACAs, auto-inhibited Ca^2+^-ATPases; CaMs, calmodulin; CMLs, calmodulin-like protein; CBLs, calcineurin B-like protein; CIPKs, CBL-interacting protein kinases; CDPKs, Ca^2+^-dependent protein kinases.

The evolutionary characteristics of potential orthologues from 15 gene families in Ca^2+^ signaling (i.e., channels, pumps, co-transporters, and sensors) were identified using 41 species across major plant lineages by conducting a comparative genetic similarity analysis (Figure 2) [7,56,147,148,149]. Remarkably, the proteins involved in Ca^2+^ influx exhibited relatively low similarity and conservation, especially compared with Ca^2+^ ATPases (ECAs, ACAs), CaMs and CDPKs, which are evolutionarily conserved in most tested species from red algae to angiosperms [145]. The interpretation of this result may indicate that high levels of uncontrolled cytosolic Ca^2+^ show direct cytotoxicity, such as precipitation with phosphates [145,150]. Therefore, it may be essential for plants to develop and diversify in their evolution and function with regards to Ca^2+^-ATPases and Ca^2+^ sensors for better adaptation to abiotic stresses. In another perspective, the gene families of Ca^2+^-permeable channels undergo some functional differentiation and complementation with respect to the properties of their NSCCs [151], which not only display significant permeability to both mono- and divalent cations [152], but also a potential preference for anions [153].

**Figure 3:**
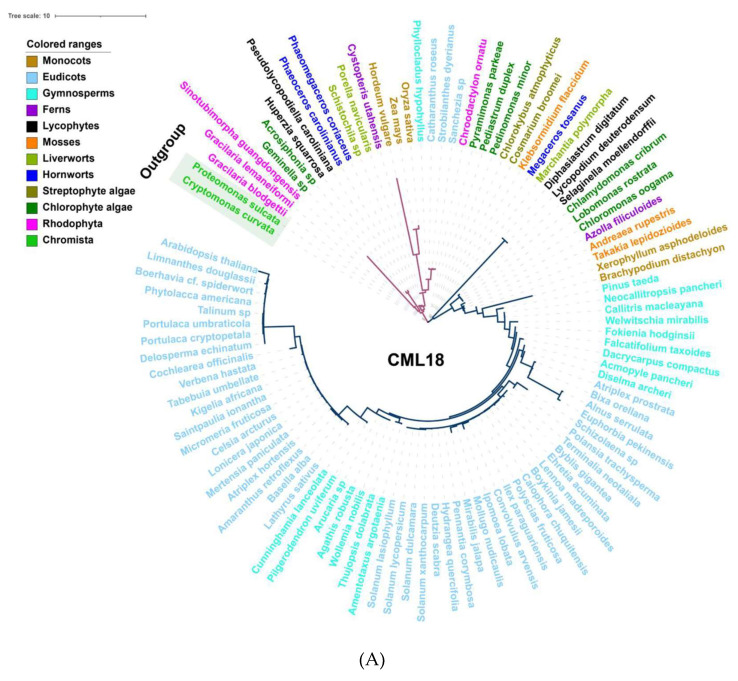

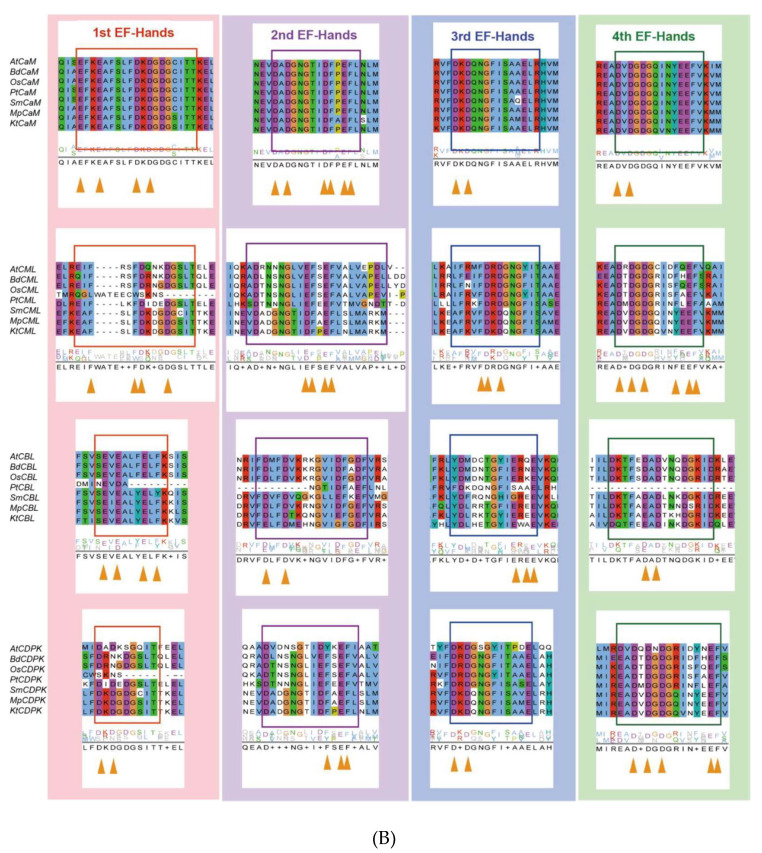
Evolutionary analysis of representative Ca^2+^ signalling proteins in the major lineages of green plants. (**A**) Phylogenetic analysis of CML18 protein in representative species of the major lineage of green plants using the OneKP and NCBI databases. The tree is constructed based on the maximum-likelihood method. Clades are indicated by different colors. Two distinctive evolutionary clades are distinguished by the purple and blue branches, respectively. (**B**) Sequence alignment of the CaM, CML, CBL, and CDPK domains among eight representative green plant species. Colored rectangles indicated 1st (red), 2nd (purple), 3rd (blue), and 4th (green) EF-hands motifs. Triangle arrows mark conserved sites. *At*, *Arabidopsis thaliana*; *Bd*, *Brachypodium distachyon*; *Os*, *Oryza sativa*; *Pt*, *Pinus taeda*; *Af*, *Azolla filiculoides*; *Sm*, *Selaginella moellendorffii*; *Mp*, *Marchantia polymorpha*; *Kf*, *Klebsormidium flaccidum*.

Among the Ca^2+^ sensors, CDPKs are found in all green plant lineages and red algae (Figure 2), which do not require the involvement of calmodulin owing to their specific structure, and so can directly self-activate and respond to Ca^2+^ signals [154]. Additionally, family- or species-specific whole genome duplication (WGD) events allow considerable variation in the numbers of CDPK proteins between plant species and clades [10,155]. In parallel, either CMLs or CaMs could be traced to basal lineages of streptophyte algae, among which CMLs exhibited a much lower protein sequence similarity compared to CaMs; however, the number of CMLs largely exceeds that of CaMs (Figure 2 and Figure S2). This can be interpreted such that sequence divergence, even in the EF hand domains, exists among the CMLs, leading to the variant structural properties of Ca^2+^-binding motifs, and resulting in the separation of the CMLs from CaMs [141,156]. CML18 (At3g03000) was clearly indicated as interacting with vacuolar Na^+^/H^+^ antiporters more than a decade ago [157]. Evolutionary and phylogenetic analysis suggested that CML18 is present in chlorophytes and Rhodophyta. However, compared with the evolutionary analysis of CaM1 reported in the previous study [106], CML18 showed two unique evolutionary clades in red algae (Figure 3A). These distinctions indicate that CMLs may have evolved earlier than CaMs and may have diversified later [145].

*TPC1,* encoding the slow vacuolar (SV) Ca^2+^ channel, is mostly found in land plants and algae with low copy numbers, but nine *TPC* genes exist in the model moss *Physcomitrella patens* (Figure 2) [158]. Plant annexins (ANNs) are fairly well-conserved in their repeat 1 or 4, whose function is in Ca^2+^-binding sites, but which lack Ca^2+^-coordinating residues in repeats 2 and 3 [136]. Consequently, ANNs showed relatively low conservation across the tested green plants (Figure 2). By contrast, CBL and their interacting kinase CIPKs resemble two flexible combinable modules possessing a Ca^2+^-binding domain and a kinase activity domain, respectively [159]. Considering that CBL forms several interaction pairs with CIPK, the number of CBLs are frequently 2~4-fold less compared to the number of CIPKs in green plants (Figure S2). The CBL–CIPK interaction relationship illustrates that the evolutionary strategies of highly specific interactions and spatiotemporal differential expression may support this dosage-balanced selection via a reduced number of gene copies in the CBL family [159,160,161].

Overall, Ca^2+^-binding domains are present from algae to angiosperms, but they are unevenly distributed, with diversity increasing with genome size, but also with lineage-specific variations [9,10]. CDPKs were quite abundant in algal species; CBLs-CIPKs significantly expanded before land colonization. The number of CaMs/CMLs proteins increased in the process of terrestrial adaptation (from straptophyte algae to bryophyte) and the extension of multicellularity (from gymnosperms to monocots and dicots; Figure S3) [12,98].

### 4.2. Linking Environmental Cues and the Evolution of Calcium-Signaling

Since the evolution of hydrophytes to terrestrial plants, Ca^2+^ signaling systems have experienced enormous expansion and diversification. Although other Ca^2+^-binding and responding modules are found, EF-hand domains are still vital in Ca^2+^ sensing systems. The EF-hand predominantly exists in CaMs, CMLs, CBL, and CDPKs [11]. Therefore, the traces of variation in their number and structure can also reflect their evolutionary process and adaptation to the environment, including abiotic stresses.

Ca^2+^ signaling components show a greater increasing rate of diversity than other proteins. CMLs are the most primitive EF-hand containing proteins, and subsequently, some CMLs evolved into CaMs, following which, CaMs may have merged with protein kinases to generate Ca^2+^-dependent kinases with diverse and distinct functions [162]. Thus, the EF-hand domains may give rise to a diversity of compositions and structures, which makes it easy to acquire new functional interactions [163]. CaMs maintain a high level of sequence conservation in evolution, with the existence of D-x-D motifs in all 1st EF-hands, 2nd EF-hands, 3rd EF-hands, and 4th EF-hands—which is consistent with previous studies (Figure 3B) [11]. However, CMLs contain only one F-D-x-D and D-x-D-x-D motif in the 3rd and 4th EF-hand, respectively; the rest of the EF-hands contain F-x2-F or E-F-x-E-F, which suggests that other residues have been substituted D (Asp) in CMLs. There are three EF-hands in CDPKs possessing D-x-D motifs, exhibiting a considerable structural similarity. Given the effects of molecular interaction mechanisms, CBLs have a greater degree of differentiation in their EF structure and possess only one D-x-D motif in their 4th EF-hand (Figure 3B). Therefore, the understanding of EF-hand-containing proteins demonstrates that duplication and loss events have occurred in the evolutionary process of EF-hand molecules, of which duplication preceded loss events [9,11].

**Figure 4:**
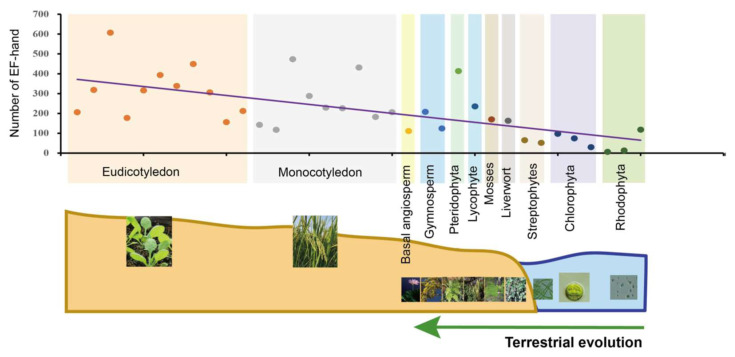
Statistics and analysis of the quantity of EF-hand domains in 35 plants and algal species. These species were classified into 11 evolutionary clades indicated by different colors. Each point in the different colors, arranged by evolutionary process, represents one species each. The same-color points are located at the same evolutionary stage. Fitted trendlines indicate that the quantity of EF-hand domains is increasing. Relevant data was integrated with superfamily databases and local retrieval was performed with the Hidden Markov Model (HMM) using hmmsearch (http://www.hmmer.org/; accessed on 13 June 2021).

CMLs, CaMs, CBLs and CDPKs account for more than one-third of all the EF-hand domains present in plant genomes [145]. The overall trend is a great and sustained increase in EF-hand domains in the evolutionary process of green plants (Figure 4), whereas the quantity of EF-hands was originally very low in early algae, indicating that Ca^2+^ sensing appeared to experience a differential expansion and functional specialization in CMLs, CaMs, CBLs and CDPKs. This scenario indicates that Ca^2+^ sensors may have acquired multiple distinct functions to break the evolutionary bottleneck via doubling and diversification. Changes in the number of Ca^2+^-sensing genes and EF-hand motifs may also be linked to abiotic stresses and the morphological complexity of plants (Figure 4). Additionally, the loss of abundance and increase of function complexity forced novel and sub-functions from a limited number of sensors [12]. CDPKs are relatively abundant in land plants and algae, and expand during terrestrial transition and/or adaptation in plants [155]; CBLs are relatively scarce (Figures S1 and S2). This may indicate that CDPKs played potential functional roles in the evolution of land plants because of their kinase activity, and the functional multiplicity of CBLs depends on their interactions with CIPKs. Moreover, the number of CaM and CML genes is majorly correlated with transitions from streptophyte algae to bryophyte (Figures S2 and S3), which differs slightly from previous reports [98,164]. This demonstrated that expansion of CaM and CMLs is congruent with aquatic plants adapting to terrestrial environments under selective pressures, such as land colonization, and harsh and variable conditions. It is worth noting that new CML classes with new biological properties were found in mostly green plant lineages [12], which is very important for future studies into the evolution of Ca^2+^ signaling in green plants.

### 4.3. Transcriptomic Analysis Reveals Ca^2+^ Regulation under Abiotic Stresses

**Figure 5:**
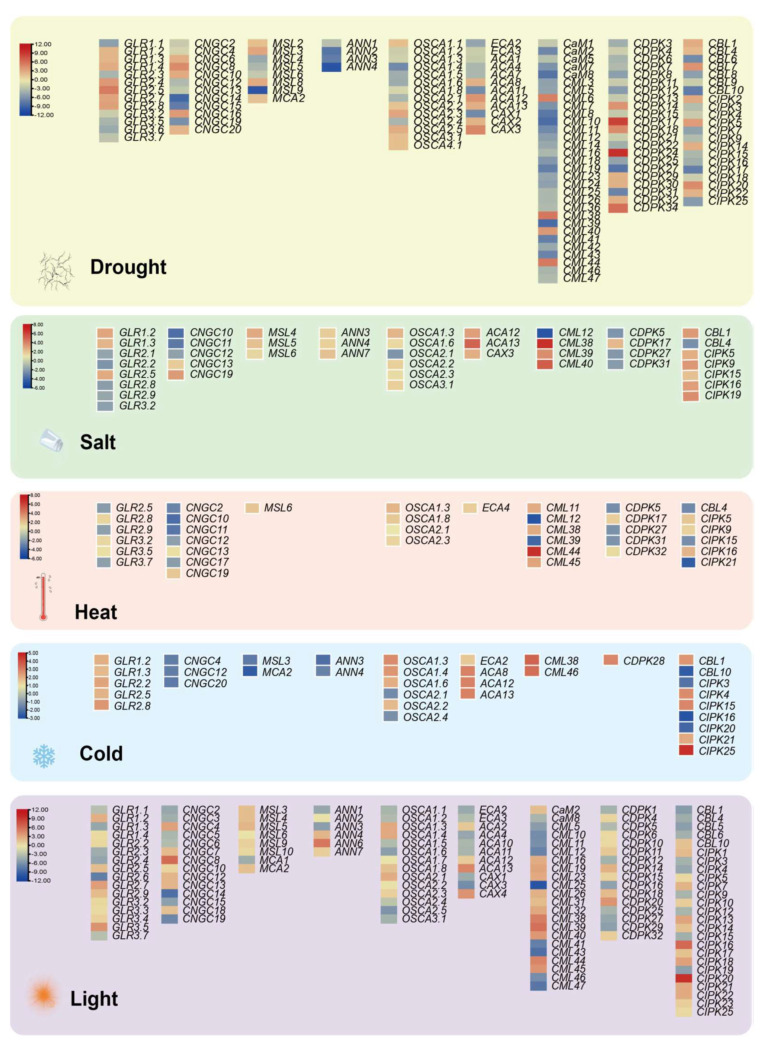
DEGs-based integrated expression profiles of Ca^2+^ singling genes in drought, salt, heat, cold and light. DEGs are filtered in the Expression Atlas databases with the following setting parameters: adjusted *p*-value < 0.001, |log_2_^[fold change]^| > 1. Red, up-regulated; Yellow, non-changed; Blue, down-regulated. The scale of the different stress modules also slightly varies.

The major plant response to abiotic stresses is mediated through gene expression patterns, leading to reconfiguration of the proteome and resulting in conferred stress tolerance. Among them, the proteins of Ca^2+^ signaling regulated by intracellular Ca^2+^ are valuable approaches to stress stimulus signals. Transcriptomic expression analysis of Ca^2+^-related genes under multiple abiotic stresses (e.g., drought, salt, heat, cold and light) were performed using the public database in *Arabidopsis* (https://www.ebi.ac.uk/gxa/home; accessed on 27 June 2021) with the following setting parameters: adjusted *p*-value < 0.001, |log_2_^[fold change]^| > 1. The differentially expressed genes (DEG) of the calcium-related gene family present with drought treatments and light conditions are more numerous, at 137 and 132, respectively (Figure 5), where the highest differentially expressed value is reached separately at 10.3 and 11.4. The remaining abiotic adversities separately contain 43 (salt), 36 (heat) and 34 (cold) DEGs, with a maximum difference value of 6.6, 7.8 and 4.6—even the annexins gene family is absent from the heat stress module (Figure 5). Meanwhile, there are seven DEGs actively modulated in all abiotic stress exposures, namely, GLR2.5, CNGC12, OSCA1.3, OSCA2.1, CML38, CIPK15, and CIPK16. Previous studies have demonstrated that the expression of GLR2.5 displays a notabe elevation under salt treatment conditions [165] and that the CIPK15 homologous gene could improve cold stress resistance in *Nicotiana tabacum* [166]. Screening by forward genetics, AtCIPK16 was identified as a mediator diminishing shoot salt accumulation and enhancing salinity tolerance in *Arabidopsis* and transgenic barley [167]. Nevertheless, research on CNGC12 has mostly focused on mediating pathogen defense and cell death without abiotic stresses [168,169]. Similarly, OSCA1.3 and CML38 are implicated in regulating plant stomatal immunity and root growth inhibition rather than in abiotic stresses responses, respectively [170,171]. Simultaneously, OSCA2.1 currently lacks direct studies providing any definitive evidence.

Certainly, several reported genes are not listed in the five modules due to different experimental condition settings and regulatory mechanisms; for example, CDPK23 is involved in the osmotic stress exerted by drought and salt stresses in *Arabidopsis* [33], and CaM was shown to shape thermotolerance through interactions with relevant kinases [54,172]. These results provide insights into the balance between the dosage effect of a gene and the compensatory mechanism of the pathway where the gene is located. Overall, investigation of calcium signaling interconnection with other secondary messengers and hormonal pathways in plants is likely to continue to be a hot topic in the evolution and molecular function of gene families in green plants, especially with the rapid increase of a large number of genome assemblies across the plant kingdom.

## 5. Concluding Remarks and Future Perspectives

Calcium has emerged as a ubiquitous second messenger between abiotic stresses and physiological responses. A large and sensitive network of Ca^2+^ signaling events has been refined in plant cells during their adaptation and interaction with the environment. Ca^2+^ channels are involved in the influx of Ca^2+^ and the regulation of Ca^2+^ gradients, Ca^2+^ sensors decode Ca^2+^ signatures to decipher the nature of the stimulus, and the signals are then transduced through the appropriate stress hormone pathways to produce a series of physiological responses (Figure 1) [9,12,173]. As a central node, the generation of Ca^2+^ spatiotemporal patterns coordinated by Ca^2+^-permeable channels, and the subsequent positional-directional interaction of downstream sensors, remain questionable. Specifically, the majority of channels do not possess strict selectivity for Ca^2+^, which suggests that other regulatory pathways may have made some concessions for Ca^2+^ flux [21]. Remarkably, the propagation of Ca^2+^ waves as mobile signals precisely integrates activities throughout plants, whose mechanisms, nevertheless, lack systematic descriptions [6]. In parallel, Ca^2+^ sensors exhibit functional redundancy, and the absence of a single sensor does not elicit significant changes in response to abiotic stress. Collectively, Ca^2+^ is implicated in crosstalk between multiple signaling pathways, coupled with phytohormone transduction, forming an intricate signal network. However, these above-mentioned questions, as challenges for scientists, still need urgent intensive research.

From an evolutionary perspective, the functional exertion of calcium-related genes is likely to have certain dosage and complementary effects. Ca^2+^ signaling displays fundamental differences to other protein evolutions in general; Ca^2+^ signaling components encompass a wide evolutionary diversity, with either conservation or variation, which are both required for developing the capability to adapt to complex and changing environments [12,108]. Furthermore, as molecules that respond directly to external stimuli, multiple Ca^2+^-sensing proteins may carry the imprints of evolutionary adaptation to abiotic stresses or break evolutionary bottlenecks in various ways. With the increasing number of sequenced green plant genomes, significant progress has been made towards our understanding of this evolutionary fundamental process, which may provide the molecular and bioinformatics background for their functional analysis in some non-model species in the evolutionarily important green plant lineages. Consequently, despite the considerable advancements in the recent research of Ca^2+^ signaling system in plants, comprehensive analysis from signal recognition to adversity response requires further exploration. We propose future research work in exploring Ca^2+^ signaling functional genes from the model plants Arabidopsis and rice to lay the foundation for improving crop abiotic stress tolerance. It is also worth investigating the evolution and functional differentiation of Ca^2+^-related gene families in abiotic stresses using the model moss *Physcomitrella patens* and other early divergent plant lineages. Finally, we should build the regulatory network of Ca^2+^-related proteins for individual cell types and integrate the potential molecular interactions between different Ca^2+^ response modules for different abiotic stresses.

## Data Availability

Not applicable.

## Supplementary Materials

The following are available online at https://www.mdpi.com/article/10.3390/ijms222212308/s1.
