# Supplementary figures and images for: Molecular Evolution of Calcium Signaling and Transport in Plant Adaptation to Abiotic Stress
Source: Int J Mol Sci. 2021 Nov 15;22(22):12308. doi: 10.3390/ijms222212308 (PMC8618852; doi:10.3390/ijms222212308)

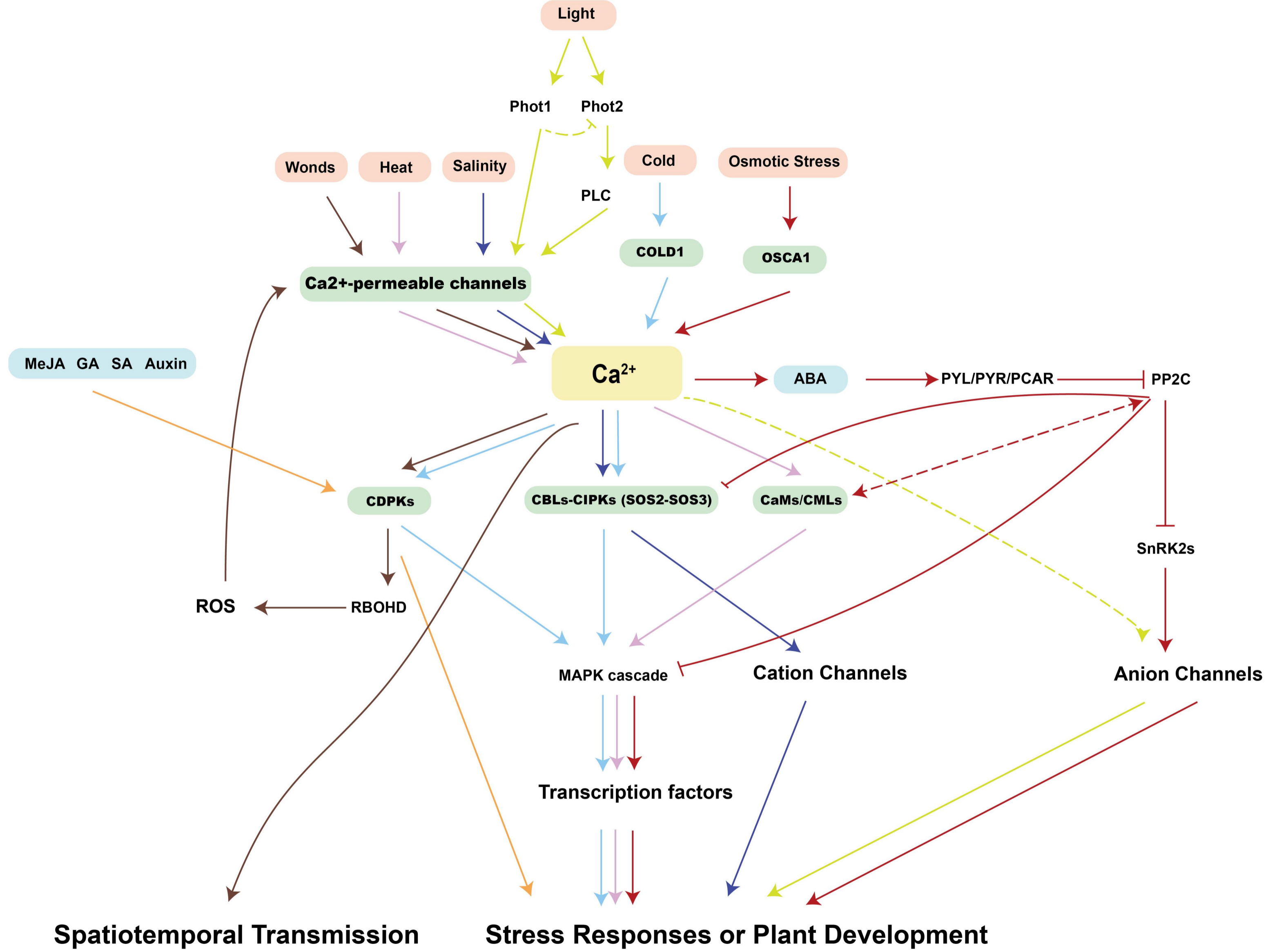

Supplement: Supplementary file 1 [file ijms-22-12308-s001.zip › Supplementary Files/Figure S1.pdf]

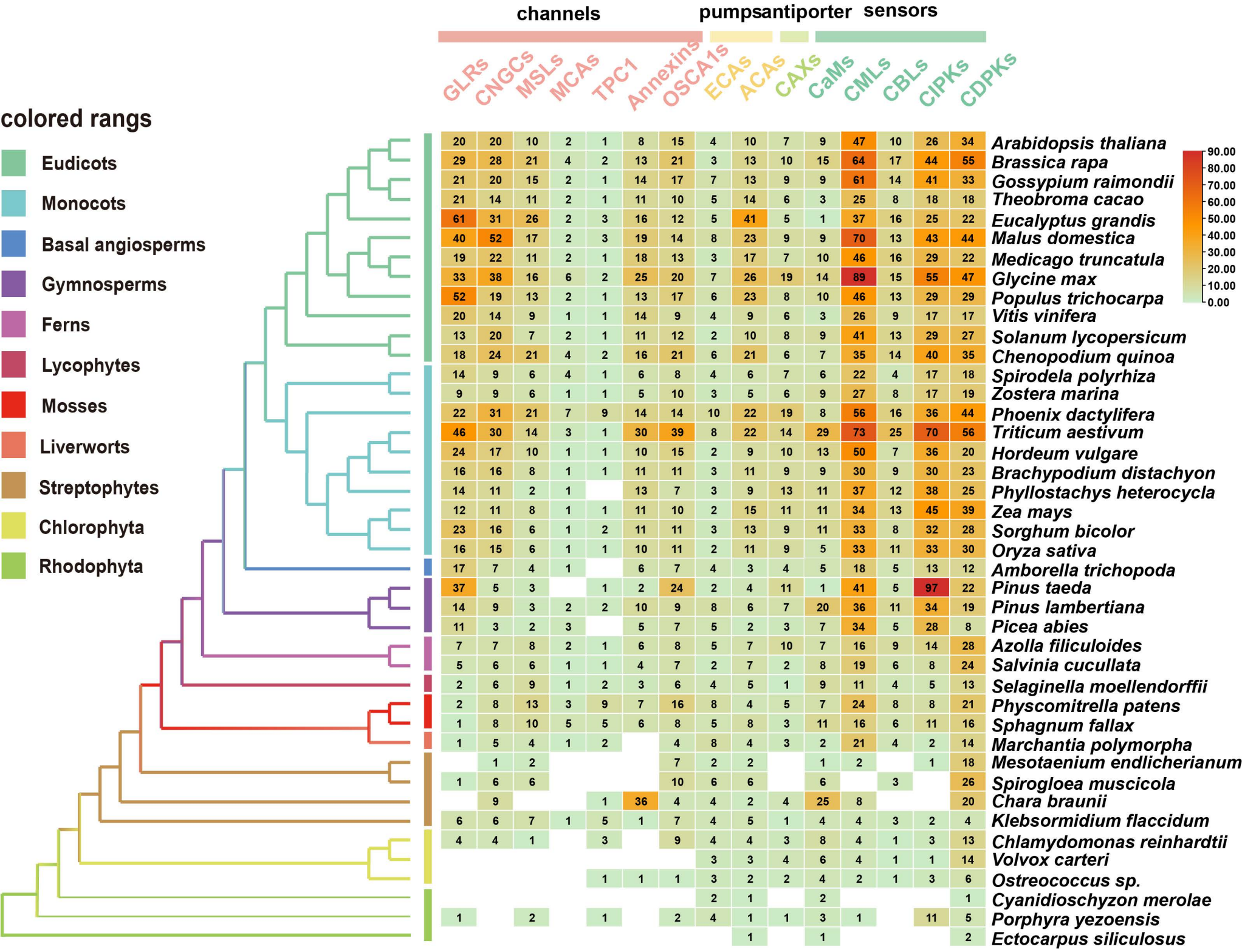

Supplement: Supplementary file 1 [file ijms-22-12308-s001.zip › Supplementary Files/Figure S2.pdf]

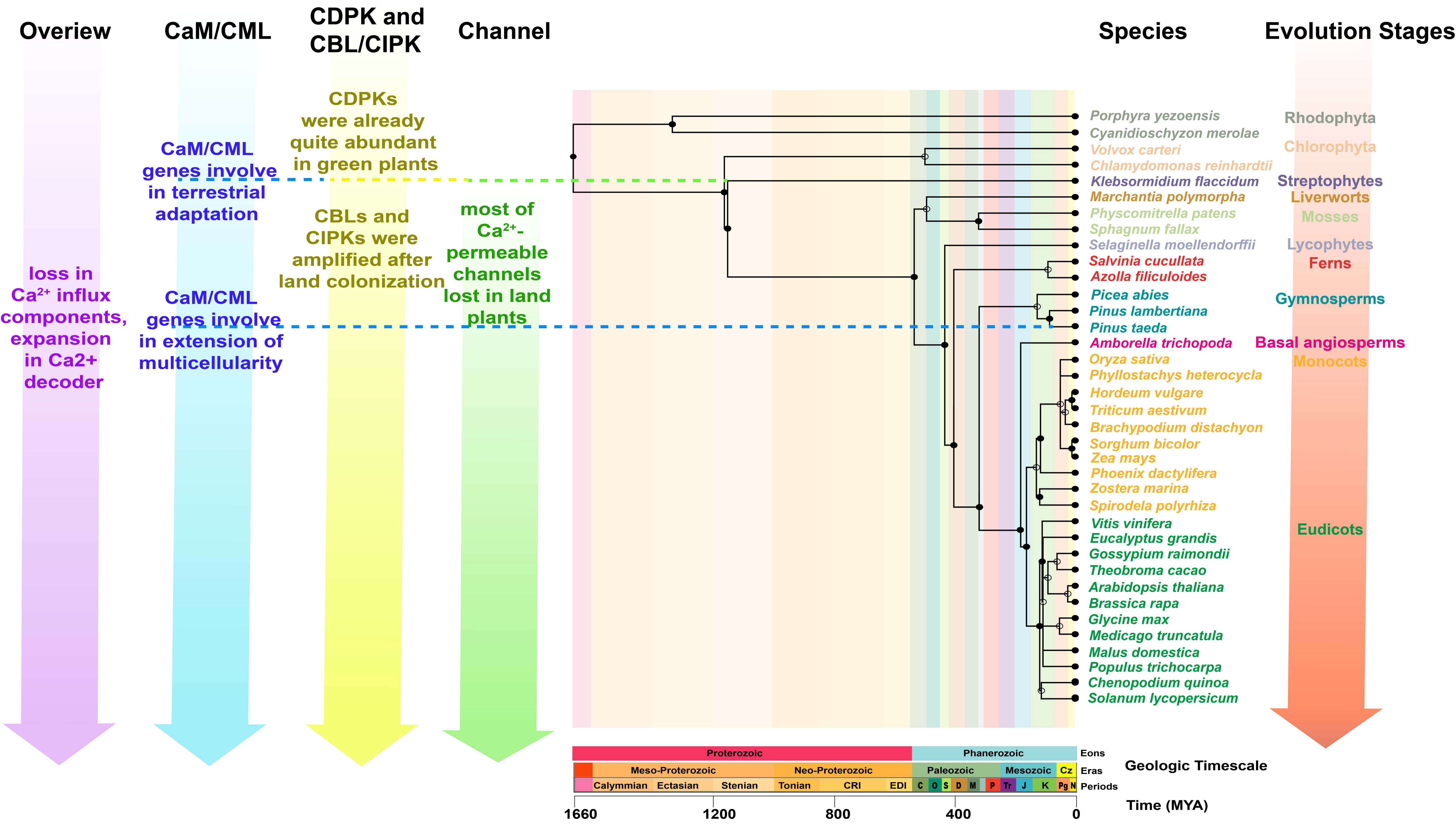

Supplement: Supplementary file 1 [file ijms-22-12308-s001.zip › Supplementary Files/Figure S3.pdf]
